# The Rise of New Alcoholic Games Among Adolescents and the Consequences in the Emergency Department: Observational Retrospective Study

**DOI:** 10.2196/pediatrics.6578

**Published:** 2018-04-27

**Authors:** Stefania Barbieri, Luca Omizzolo, Alberto Tredese, Gianna Vettore, Alberto Calaon, Astrid Ursula Behr, Rossella Snenghi, Massimo Montisci, Rosa Maria Gaudio, Andrea Paoli, Vincenzo Pietrantonio, Jacopo Santi, Daniele Donato, Giovanni Carretta, Annalisa Dolcet, Paolo Feltracco

**Affiliations:** ^1^ Department of Urgent and Emergency Care University of Padova Padova Italy; ^2^ Preventive Medicine and Risk Assessment University of Ferrara Ferrara Italy; ^3^ Forensic Medicine and Toxicology University of Ferrara Ferrara Italy; ^4^ Department of Legal Medicine University of Padova Padova Italy; ^5^ Department of Directional Hospital Management Padova General Hospital Padova Italy; ^6^ Surgery Department King's College Hospital NHS Foundation Trust London United Kingdom

**Keywords:** adolescent, neknomination, binge drinking, alcoholic games, social network

## Abstract

**Background:**

The links between the internet and teenager behavior are difficult situations to control and may lead to the development of new and excessive methods of drinking alcohol during alcoholic games. Findings indicate that reported cases are very useful sources for better understanding of alcoholic games, yielding successful measures promoting health among adolescents. Admittance of adolescents to hospital emergency departments (EDs) after consumption of excessive amounts of alcohol has become the norm in developed countries. The harmful effects of acute alcohol abuse are reported in this paper.

**Objective:**

The aim of this work was to investigate the close connections between new drinking behaviors among adolescents and study the increase in new alcoholic games, together with the challenges that cause acute alcohol intoxication, the influence of the internet and social networks, and their consequences for public health services.

**Methods:**

Data came from prehospital and intrahospital admissions attributable to alcohol consumption. From 2013 to 2015, 3742 patients were admitted to EDs due to acute alcohol intoxication: 830 of them were aged 15 to 30 years, and 225 were adolescents and young adults between 15 and 20 years who had been playing alcoholic games. Retrospectively, diagnostic data associated with extrahospital anamneses were selected by one of the hospital management information systems, Qlik. As a result of our previous experience, questionnaires and face-to-face interviews were performed at a later stage, when a clinical audit for intoxicated adolescent patients was described, with the overall goal of establishing a potential methodological workflow and adding important information to research carried out so far.

**Results:**

Between 2013 and 2015, 830 young patients aged 15 to 30 years were admitted to EDs for acute alcohol intoxication. About 20% (166/830) of the sample confirmed that they had drunk more than 5 alcoholic units within 2 hours twice during the past 30 days as a result of binge drinking. Referring to new alcoholic games, 41% of the sample stated that they knew what neknomination is and also that at least one of their friends had accepted this challenge, describing symptoms such as vomiting, headache, altered behavior, increased talkativeness, and sociability. The median value of the weighted average cost of the diagnosis-related group relating to interventions provided by hospitals was the same for both genders, €46,091 (US $56,497; minimum €17,349 and maximum €46,091).

**Conclusions:**

Drinking games encourage young people to consume large quantities of alcohol within a short period of time putting them at risk of alcohol poisoning, which can potentially lead to accidental injuries, unsafe sex, suicide, sexual assault, and traffic accidents. The spread of these games through the internet and social networks is becoming a serious health problem facing physicians and medical professionals every day, especially in the ED; for this reason, it is necessary to be aware of the risks represented by such behaviors in order to recognize and identify preliminary symptoms and develop useful prevention programs. The strategic role of emergency services is to monitor and define the problem right from the start in order to control the epidemic, support planning, coordinate the delivery of assistance in the emergency phase, and provide medical education. Hospital-based interdisciplinary health care researchers collected specific data on hazardous drinking practices linked to evaluation of increased alcohol-related consequences and cases admitted to the ED.

## Introduction

**Figure 1:**
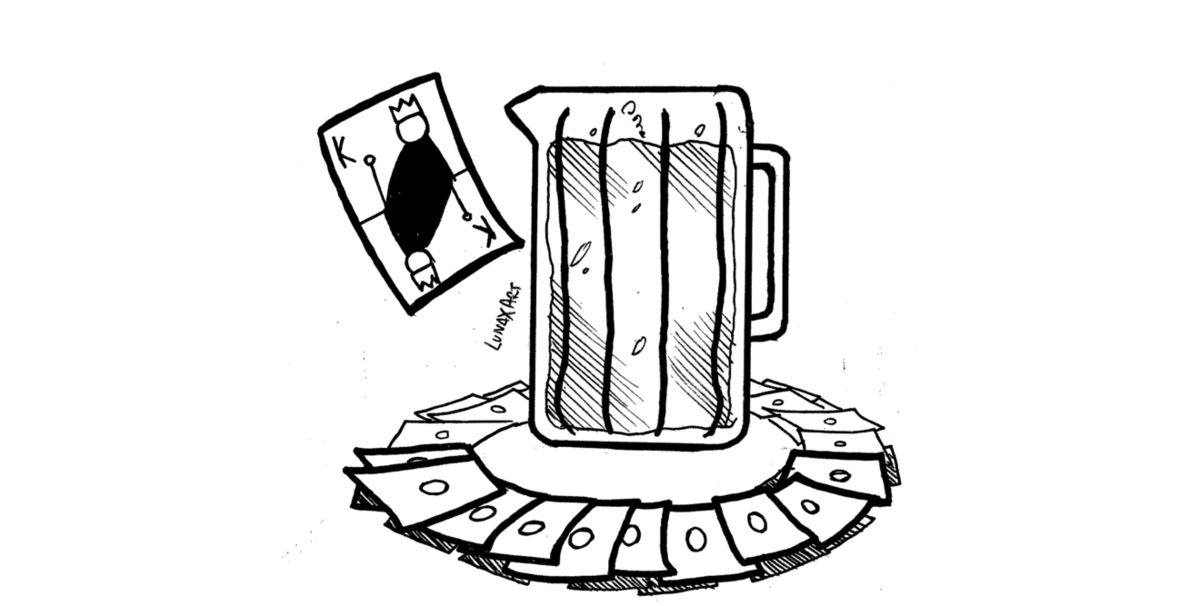
King game.

**Figure 2:**
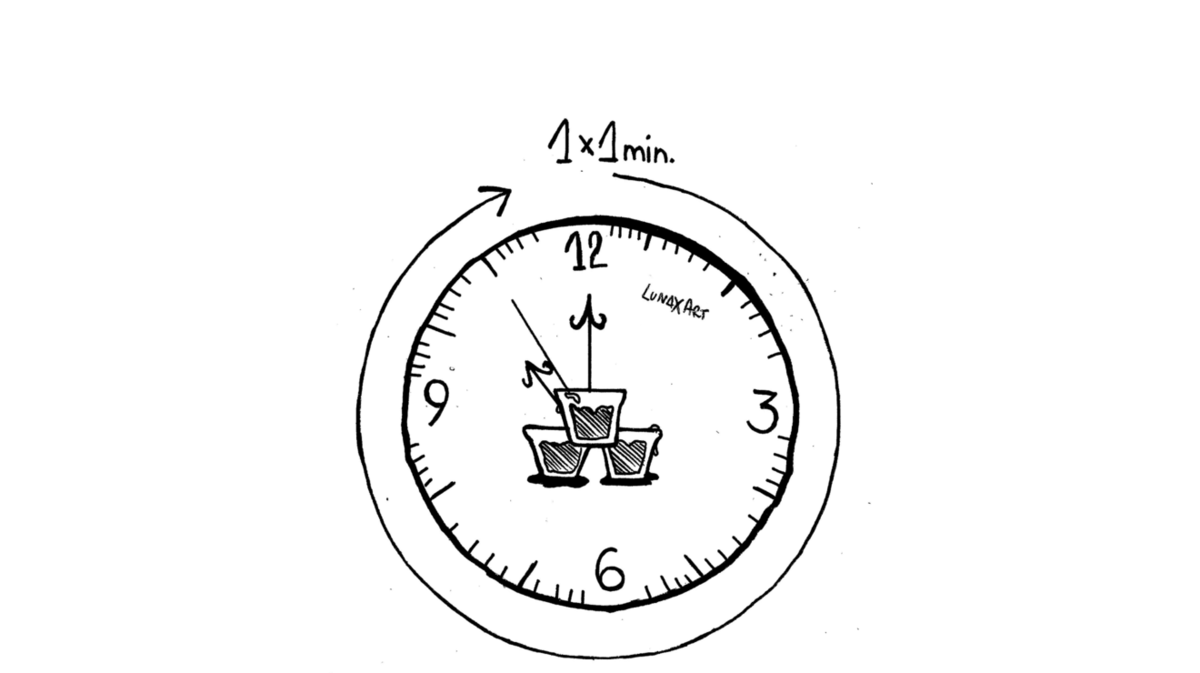
Power hour.

**Figure 3:**
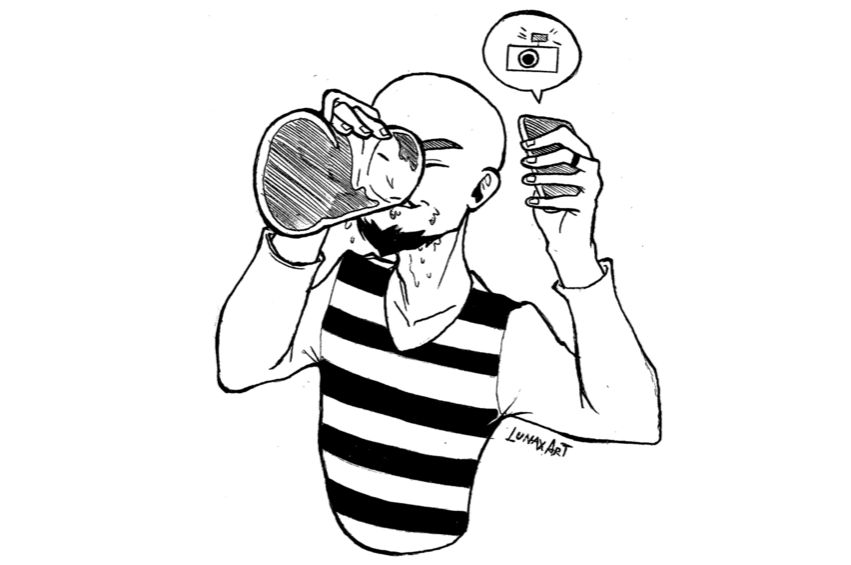
Neknomination.

**Figure 4:**
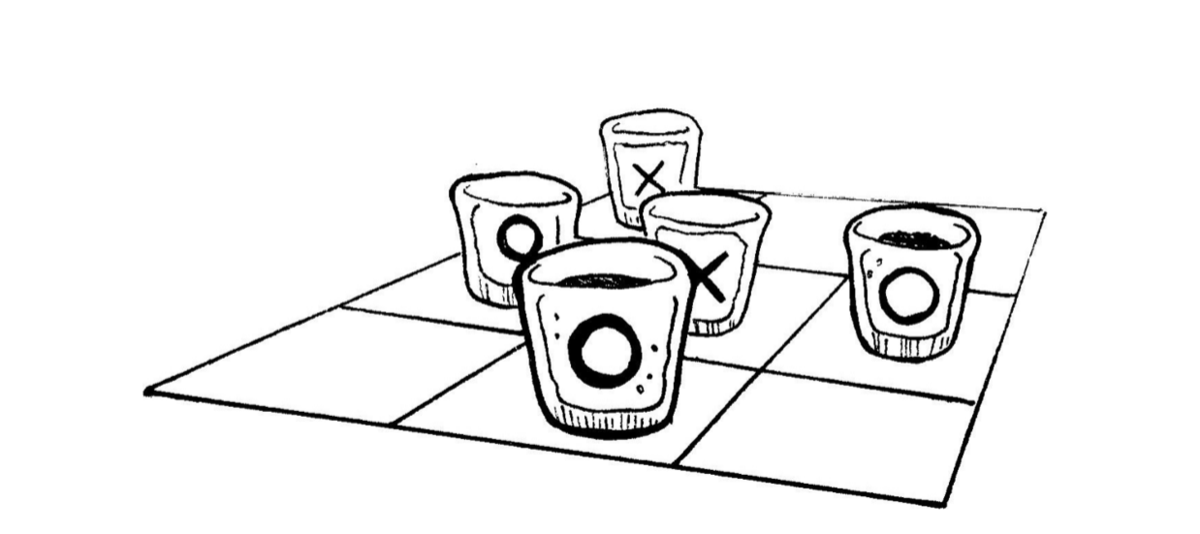
Tris shots.

**Figure 5:**
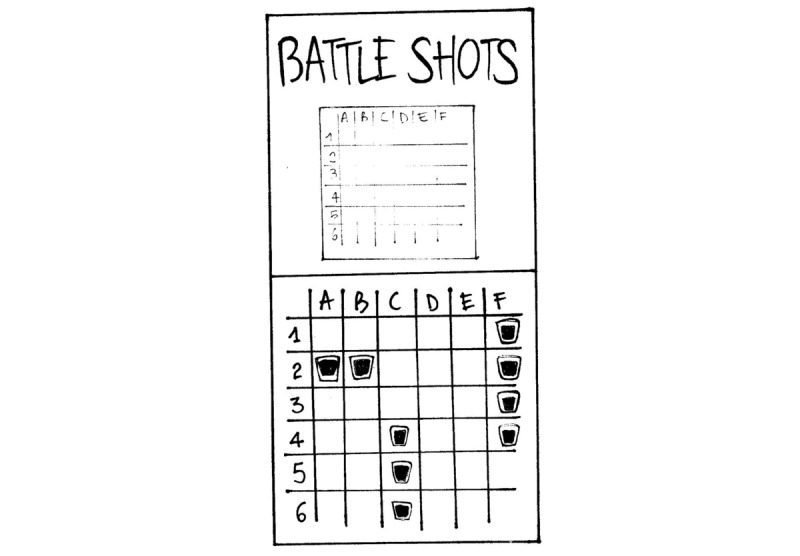
Battle shots.

**Figure 6:**
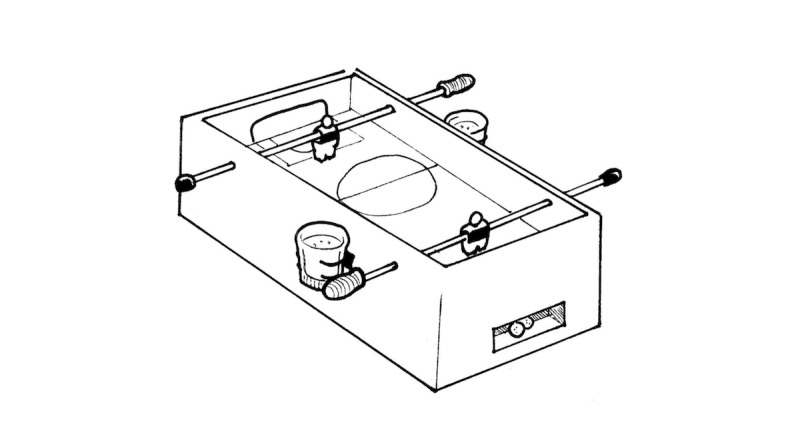
Table football.

**Figure 7:**
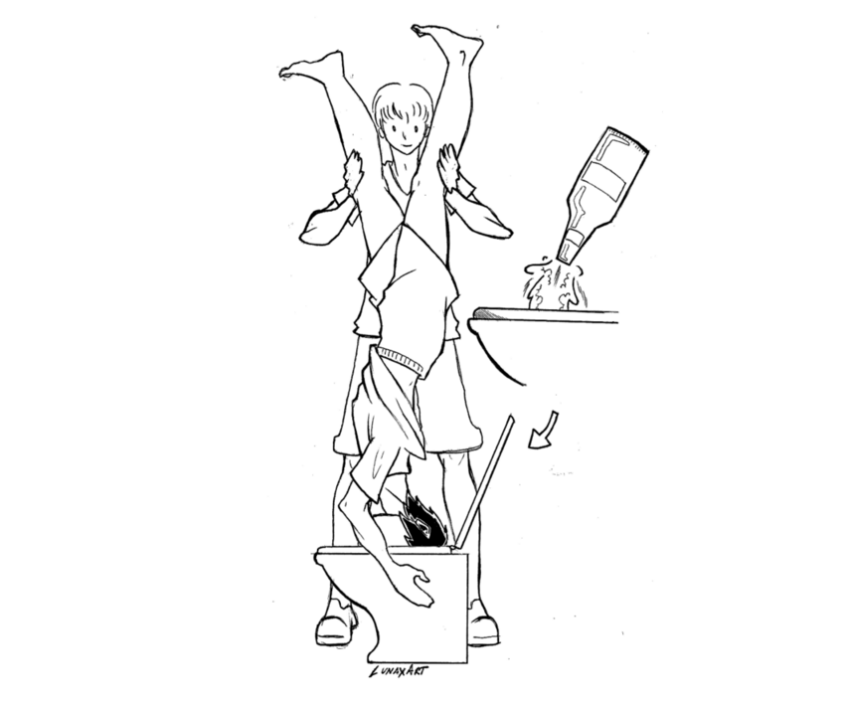
Neknomination 2.

**Figure 8:**
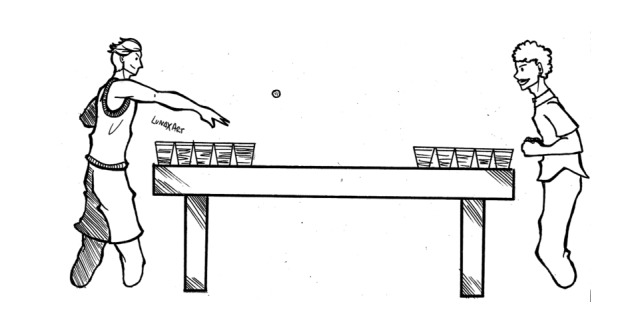
Beer Pong.

Alcohol intoxication in adolescents is associated with the main causes of death and serious injury (ie, motor vehicle, bicycle, and pedestrian accidents and suicide), but binge episodes and drinking levels that represent few problems for adults may be dangerous for adolescents. There are many projects investigating the Italian and European problem of alcohol consumption among young people [1], and some alcohol prevention programs are targeted to educational classroom interventions to encourage positive behavioral changes, inform and educate parents and teachers about the spread of new alcohol consumption modes among adolescents, and inform clinicians about the risk and impact in emergency medicine. The strategic role of emergency medicine is to define and monitor the problem as soon as it appears and then limit its spread. The aims of this work were to investigate the connection among new methods of alcoholic consumption, the spread of new alcoholic games among adolescents, and the influence of the internet and social networks and their consequences for public health in terms of numbers of accesses to emergency departments (EDs), the length of hospitalization, and the costs of these services for the Italian health system. These cases may provide important information allowing hospital personnel to recognize and identify the following themes that emerged from our analysis: (1) learning about patients’ perspectives by examining their particular cases, (2) knowledge of a critical approach to one’s own clinical practice, and (3) perception of enhanced evidence-based practice and shared decision making when intoxicated teenagers are admitted to the ED. Future surveys should identify potential markers of problematic use of social networks, including identification of groups at risk of abuse, and should aim to establish potential prevention strategies in terms of awareness and education that could help to avoid potentially fatal episodes. Little evidence is available, despite the costs of alcoholic game-related risks and the problems of teenagers arriving at the ED. Alcoholic games are often not recognized or identified in the ED, suggesting the need to improve the general education of both patients and physicians. Drinking games, prepartying (ie, drinking before going to a social event), heavy episodic drinking, and alcohol-related problems in students in secondary education have been reported in several previous works [2-8]. The descriptive longitudinal study by D’Amico et al [9] analyzed the various habits of adolescents and identified a close connection between alcohol abuse and consumption of other substances such as tobacco and marijuana, and its authors demonstrated a greater propensity to alcohol abuse in people of Hispanic and African American descent. The authors then stated that the general assumption of most descriptive models of peer pressure is based on adolescents’ perceptions of the consumption of alcoholic beverages by their friends, identified as a predictor of long-term substance abuse in adolescents and preadolescents [9]. According to Borsari et al [8], Poliziotto et al [10], and Zamboanga et al [3], binge drinking is associated with secondary education students who were admitted to hospitals with alcohol-related problems, but there are very few reports about alcohol problems due to alcoholic games in adolescents. In recent years, adolescents have been participating in extreme alcoholic games for a variety of reasons (having fun, the idea of competition, etc) [11-13]. Health care intervention regarding alcohol intoxication in adolescents is a critical component in the implementation process in EDs [13-15]. Alcohol-derived injuries and fatalities due to alcoholic games are a serious problem for public health and medical science. Adolescents admitted to EDs for acute alcohol consumption represent an emerging public health problem, and social media games encourage alcohol abuse with alcoholic games, especially king game (Figure 1), power hour (Figure 2), neknomination (Figure 3), tris shots (Figure 4), battle shots (Figure 5), table football (Figure 6), neknomination 2 (Figure 7), and beer pong (Figure 8). Neknomination is a new form of a social online drinking game in which participants film themselves while drinking a pint of alcohol or spirits in one gulp and upload the resulting videos on the Web; the appointed person must complete this task within 24 hours [11,15,16]. Alcoholic games should not be confused with binge drinking because of the different characteristics of clinical prehospital assessment, definition, epidemiology, and risk factors. Clinical data epidemiology and risk factors appearing on emergency medical service charts represent a significant opportunity for research that could lead to effective solutions. The aim of this research was to show health professionals how to analyze the anamnestic data collected in prehospital charts and diagnosis-related group (DRG) codes according to all coded diagnoses, procedures, and ED development technologies regarding adolescent alcohol consumption patterns during alcoholic games. The importance of considering alcoholic games as a cause of severe and acute alcohol use is shown by the fact that accurate anamneses can reveal a patient’s circumstances both prehospital and in the ED. Although emergency medical service data focus on quantitative events whereas clinical data regarding adolescent and alcoholic games mainly aim at supporting emergency medical service policy decisions and related research, they also represent an opportunity to share best practices in order to find valuable information in the data collected. The important relationships among standardized prehospital care data (clinical, management, administration), research, and clinical decision making is an opportunity to change perceptions in large-scale research.

## Methods

This research is an observational retrospective study conducted at the University Hospital of Padova, Italy. Adolescents and young adults aged 15 to 20 years who accessed the ED of Padova’s main hospital answered questionnaires about a particular method of drinking and acute alcohol intoxication. The link between the internet and teenagers is little known and difficult to check, but it may lead to the development of new methods of consumption of alcoholic drinks. Among the intoxicated adolescents reaching EDs, only the cases in which they had posted videos on the Web while they were drinking “several pints of an alcoholic drink in one gulp” were selected. From 2013 to 2015, 3742 patients were admitted to the ED for acute alcohol intoxication, 830 of whom were 15 to 30 years old, and 225 of whom were adolescents and young adults between age 15 and 20 years and had been playing alcoholic games. Acute alcohol intoxication was confirmed according to a blood alcohol content (BAC) test using the alcohol dehydrogenate method and ultraviolet spectrophotometer in the laboratory of the same hospital as the ED before inclusion in the sample. Data were entered in Office Excel 2007 (Microsoft Corp) spreadsheets and analyzed with SAS version 9.2 (SAS Institute Inc) for Windows. Categorical variables (gender, diagnosis at end of observation period, day of the week, BAC, age) included the number of patients per category, and age was used to classify subgroups (15 to 20 years, 21 to 25 years, and 26 to 30 years). The variable blood alcohol level was divided into classes according to article 5 of Italian decree number 151/2003, converted with law number 214 August 1, 2003 (<0.5 g/L, 0.5 to 0.8 g/L, 0.8 to 1.5 g/L, >1.5 g/L). The chi-square test or Fisher exact test were used to evaluate the association between categorical variables. Significant *P* values were less than or equal to .05; minimum, maximum, median, and quantiles were calculated for continuous asymmetric data (duration of hospitalization, weight standard DGR) and were then compared between genders using a Wilcoxon nonparametric test. We selected 225 patients belonging to the 15- to 20-year age group because they were considered the main beneficiaries of new trends about alcohol consumption.

## Results

This study reviewed all prehospital ambulance records in Padova together with ED information sources for 2013 to 2015 regarding cases of acute alcoholic poisoning of teenagers due to alcoholic game challenges via the Web; the medical and nursing questions were asked for the purposes of this report. Previous clinical audits on alcoholic games and health-related internet problems in adolescent and young patients in an outpatient setting (lessons at medical school) are published elsewhere [11-16].

There is little information on the epidemiology and etiology of alcoholic games in adolescents in EDs and prehospital settings; the results guided health information ED systems through development and implementation in real-world hospital settings. Our implementation strategy was to develop a multifaceted intervention regarding knowledge, skills, and attitudes among underage adolescents. Diagnosis at entry, characteristics (gender, age, nationality), clinical anamnestic data required by ED doctors, BAC during the emergency phase, and in-hospital monitoring all indicate the existence of specific categories of acute alcohol use linked with challenges via the internet. Anamnestic data confirmed acute intoxication following alcoholic games. This research aims at focusing the attention of medical professionals on a growing phenomenon among adolescents—the rapid development of the internet and social networks and their consequences [14-16]. From 2013 to 2015, questionnaires were collected when patients entered the ED or later if they were comatose. Data collection of raw material describing the representation of facts in prehospital care associated with more information gathered in the clinical setting (results of questionnaires) influences decision making. In order to understand precisely how dominant the role of social media is, patients were asked to express their opinion on the importance of the influence of the Web among their peers. Evaluation of the impact of social networks on the health and daily lives of adolescents had previously been assessed with questionnaires prepared by a multidisciplinary research group with Spanish colleagues, partly supplemented by some questions pertaining to smoking and alcohol consumption. ED staff completed questionnaires regarding alcoholic games and the resulting acute intoxication. The alcoholic substances involved are in fact poisons. Increasing providers’ knowledge of patients arriving at EDs with toxicological syndromes should indirectly improve the development of local diagnostic and management protocols and guide clinical and educational initiatives to reduce morbidity due to toxicological disease after alcohol challenges on the Web. The distribution of gender and alcoholic consumption by age group in a target population is listed in Table 1.

A total of 225 of patients aged 15 to 20 years were admitted in the ED. One important factor to consider is the legal drinking age in Italy is 18 years. Clinical presentation, prompt evaluation of acute alcohol intoxication, and appropriate management are essential to ensure optimal outcomes—in particular, advanced airway management in critical prehospital adolescents can indicate the severity of the patient’s condition. The most frequently reported symptoms are unconsciousness or reduced consciousness requiring intubation by emergency medical service physicians in a prehospital setting so that patients then need further treatment and transport to EDs. The varying severity of clinical symptoms includes impaired levels of consciousness associated with seizures, slurred speech, lack of coordination, unsteady gait, nystagmus, attention or memory impairment, tremors, altered behavior, abnormal reflexes, altered perception of reality, absence of perception, and sense of death (Table 2).

Of the 225 patients, 71 stated that they had drunk great quantities of alcohol while playing alcoholic games: 14 neknomination (19.7%), 4 vodka eyeballing (5.6%), and 53 (74.6%) other alcoholic games.

Of the 830 patients analyzed from 2013 to 2015, of special interest are the 225 in the 15- to 20-year age group because they are the ones mainly exposed to new modes of high-alcohol drinking (Table 1, Table 3).

**Table 1:** Distribution of gender and alcohol consumption by age group in a target population between 2013 and 2015.

Characteristic		Age group					
		15 to 20 years, n (%)		21 to 25 years, n (%)		26 to 30 years, n (%)	
**Gender**							
	Male		135 (16.2)		186 (22.4)		219 (26.4)
	Female		90 (10.8)		126 (15.2)		74 (8.9)
**Blood alcohol content (g/L)**							
	0 to 0.5		13 (1.6)		23 (2.8)		22 (2.7)
	0.5 to 0.8		4 (0.5)		7 (0.8)		12 (1.4)
	0.8 to 1.5		36 (4.3)		20 (2.4)		27 (3.3)
	>1.5		172 (20.7)		262 (31.6)		232 (28.0)

**Table 2:** Symptoms of youth admitted to emergency department by frequency.

Year	Intoxication, n	Syncope, n	Trauma, n	Coma, n
2013	63	3	12	3
2014	73	3	22	5
2015	29	0	8	4

**Table 3:** Blood alcohol content of youth admitted to emergency department.

Blood alcohol content	Year		
	2013	2014	2015
0 to 0.5 g/L	6	7	0
0.5 to 0.8 g/L	2	2	0
0.8 to 1.5 g/L	12	16	8

Analysis and knowledge of context and proper presentation of facts and data gleaned from past experience are necessary to improve planning. In particular, researchers should focus on determinants of new modes of binge drinking to investigate acute alcohol assumption as a behavioral consequence of alcohol game challenges. The questions listed in the data survey also examine exactly where these behaviors took place (eg, at parties, clubs, or pubs or even at home, where the risk of drinking alcohol in large quantities is higher). To identify adolescents’ worst habits, they were asked if they were aware of similar behavior among their friends or family members. To assess the consequences of substance abuse from a clinical viewpoint, adolescents were also asked if they knew about new games such as neknomination and vodka eyeballing, if they had ever tried them, and if they regretted doing so. Demographically, 15- to 20-year-olds fall into the group with the highest number of orders for BACs at EDs. Hospital discharge folders and mode of access to EDs, diagnosis on entry, characteristics (gender, age, nationality), circumstances, and examinations required by ED doctors and in-hospital monitoring were all examined (ED short observation monitoring, hospitalization, operating theater, transfer to intensive care) along with BACs. We used the hospital database, Galileo DB 41 version 1.4.3.107, to compare missing data, including anamneses (provided directly or by friends) and symptoms attributable to alcohol consumption (vomiting; altered reflexes; impaired vision; perception of shapes, colors, or sizes; serious impairment of physical or mental condition; marked difficulty in standing or walking; hallucinations; cessation of reflexes; incontinence; and coma). For patients admitted to short stay observation in the ED (97/566, 17.1%), the average hospitalization time was 6 hours. Thirty patients were admitted for trauma, and their median time of hospitalization was 11 days with a 2:1 ratio between males and females and an average DRG weight of €46,000 (US $56,497) for both genders. The median value of the average DRG weight relative to hospital interventions was the same for both genders (ie, €46,091 (minimum €17,349 and maximum €46,091).

## Discussion

### Principal Findings

Results showed that it is possible to support planning, coordinate the delivery of assistance, and provide medical education in EDs. Since accurate information is essential for good decision making, a variety of initiatives, programs, and projects have led to noteworthy improvements in educational opportunities—not only for ED personnel but also for pediatricians and public health personnel. Clinicians help with treatments, and playing an essential role is important to ensure a source of health care services and motivational interviewing (which was originally developed to treat and understand alcohol abuse but can also be applied successfully to our understanding of changes in alcohol-induced behavior). This study may be viewed as the outcome of various activities: the administration of an appropriate and detailed description of attitudes about alcohol consumption among young people, systematic analysis of the related literature, and examination of hospitalization procedures for acute alcohol intoxication at the ED (an aspect which is still poorly understood). Quantification of cases and knowledge of the clinical implications of the various modes of acute alcohol intoxication among adolescents are the focal points for developing methods to deal with these new ways of consuming alcohol. Further alcohol-related social consequences include traffic and domestic accidents, legal and money problems, and gambling. Alcohol abuse can also lead to the development of dangerous behaviors such as increased consumption, unprotected sex (increasing sexually transmitted diseases and unwanted pregnancies), and driving under the influence of alcohol [5-10]. New trends such as neknomination and binge drinking are leading to higher numbers of episodes of drunkenness [16,17]. The current scientific literature contains several articles on alcohol abuse and binge drinking patterns [16-20], but only a few of them deal with alcohol consumption in adolescence and there are few data about the new ways of drinking such as neknomination or vodka eyeballing [16]. An examination of scientific journals in PubMed and Embase databases did not reveal any significant publications on the relationship between clinical sequelae due to neknomination or access to EDs as a result of alcoholic games. Alcoholic games should not be confused with binge drinking, because the characteristics of prehospital clinical assessment, definition, epidemiology, risk factors, and the Web connection all differ. Communications with the public, through the media and in schools, play a key role in gaining reliable information and prompt cooperation, thus limiting the effects of the problem. It has become necessary to invest in the development of specific strategies to stop new drinking habits in adolescents, promoting a healthy lifestyle, providing different access to care pathways, and developing closer cooperation with general practitioners. The excessive and sometimes pathological use of the internet is currently rising among young people in many industrialized countries in Asia, North America, and Europe. It has recently become a serious public health problem, and many authors have started writing about internet addiction. It is estimated that 95% of adolescents connect online every day in different places and with different devices: computers, mobile phones, smartphones, tablets, or e-book readers. The effects of social networks on the health of adolescents and, in general, the influence of this lifestyle are controversial, especially when teenagers have become more independent in their academic and social decisions because they are increasingly exposed to social media trends. While this may seem harmless, it can profoundly affect their behavior. One study examined the MySpace profiles of 400 adolescents and found that 56% of them contained references to alcohol and 49% explicitly required drinking alcohol [21]. Young people today are increasingly inclined to take and share selfies regularly on social networks such as Twitter, YouTube, and Facebook, where all users are represented by their profiles. It has been observed that the internet can significantly influence behaviors and forms of communication among peers. YouTube, which has been the property of Google since 2006, is currently the most popular video-sharing site in the world, with over 1 billion users per month. Some of the most popular videos on YouTube are music videos, which generate millions of views and comments. However, YouTube has also produced material which may be highly subject to misguided interpretation by users. Although some viewers may be sufficiently well informed to watch videos or advertising with a sense of detachment and a critical spirit, others may be deeply influenced by the spirit of escape, fun, and thrill described in some videos [22]. The real success of social networks lies in the ease of communication among teenagers outside the ambit of school, family, or friends; however, thanks to the virtual network, this now extends worldwide and allows anyone to share photos, videos, or status. Unfortunately, this attitude can also lead to peer pressure and pathological conditioning adversely affecting teenagers’ behavior, driving them toward potentially reckless behavior [23]. It is becoming clear that emulating dangerous acts is spreading rapidly all over the world due to the widespread use of social networks (ie, a viral phenomenon). However, little attention is paid to this aspect, although technology companies continue to create apps for smartphones and tablets and adolescents continue to emulate more and more behaviors on the Web. For example, Klout is a social networking service which offers customized statistical analysis on social media. In particular, it estimates users’ influence through a Klout Score (0-100) giving the degree of interaction of those users’ profiles on popular sites such as Twitter, Facebook, Google Plus, LinkedIn, and Foursquare. This influence derives from the amplitude of network users, the content generated, and the feedback level obtained. Initially, beer was mainly used in alcohol challenges, but the need to dare and be more popular soon brought teenagers to compete in these games with any alcoholic beverage available. The data collected through this study reflect the importance of dealing with the behaviors and health of young people who will be tomorrow’s adults [16-17]. It is therefore necessary to improve their living environment with an integrated approach and proper policies to prevent and combat tobacco consumption, obesity, physical inactivity, and abuse of alcohol and other substances. The importance of these sources of influence becomes evident when we examine the various psychophysical changes typical of adolescence (ie, they must be evaluated by medical professionals, because they have a significant impact on the health not only of teenagers themselves but also on society as a whole). Emerging research finds many factors that contribute to excessive alcohol abuse, despite a minimum legal drinking age. Variables including television and internet time and exposure to alcohol brands in movies indicate that about 90% of the alcohol consumed by people younger than 21 years is deliberately drunk so as to reach a BAC of over 0.08% [24-26]. Clapp et al [27] report that underage alcoholic game players obtain alcohol from young adults of legal drinking age and are under severe pressure to take part in neknomination games: in detail, nonhabitual players were invited to take part in a neknomination game or another online drinking game and then pressed to upload a video of themselves while downing that drink. The authors reported that binge drinking is a social experience of drinking, whereas neknomination only involves a person who drinks alone. The quantity of alcohol consumed during these events is very high, and participants suffer the same effects of binge drinking, since the game only lasts a few minutes. There are many risk factors related to alcohol abuse by young people, and conclusions are often in conflict in the literature: a person’s age when they first experience the taste of alcohol and the context in which this happens seem to be linked to the influence of social networks, although with different methods and rules. Another cause of underage drinking is the marketing of alcoholic beverages not only on the Web but in many advertisements, and also, according to the social influence model, highlighted as social norms and life skills. Some restaurants have special discounts during happy hour (one beer or cocktail costs half the normal price) which promote excessive drinking because the intake of a large amount of alcohol occurs in a short period of time. Neknomination, happy hour parties, and later online postings of videos seem to be socially accepted to contemporaries. In Italy, happy hour is a social drinking activity like pregaming (prepartying, pre-funking) for high school and pre-university students, whereas neknomination generally takes place at home and involves younger people. Very little is known about this risky drinking behavior linked with alcoholic games and challenge; here, we consider only teenagers with high levels of alcohol who were admitted to an ED. Despite available studies and research on alcoholic games, our aim is a critical analysis of medical records through an audit which may be useful in changing clinical practice regarding alcoholic games and taking actions to improve practice reflecting variations in clinical presentation and patient characteristics. The tendency to drink too many alcoholic drinks after Web challenges and alcoholic games suggests future research in this area on prevention and interventions, focusing specifically on the dangers. The sample described in this work consisted of adolescents admitted to EDs and the alternative drinking experiences by university-level students described in previous studies or binge drinkers; the drinking behaviors were stimulated by online alcoholic games and have sometimes been associated with frequent changing of sexual partners, but future work may consider distinguishing adolescent nondrinkers from adolescent drinkers, school characteristics, and lifestyle factors. Recent Italian data report harmful uses of alcohol in 6.3% of Italians [28] and a higher rate (13.2%) in non-Italians. In a 6-year retrospective work, Majori et al [29] reported 1547 patients (aged 16 years and older) diagnosed with acute alcohol intoxication in the hospital of Verona, Northern Italy. The use of alcohol has been influenced and promoted by millions of euros of investments in marketing, advertising, and sponsorships aimed at encouraging its consumption. Although these campaigns do not encourage hazardous and harmful alcohol consumption, harmful phenomena are increased by the use of technology and social media. The incidence of acute intoxication represents 10.0/1000 of admittances to the ED, of which about 57% are alcohol-related (ie, between 0.6% and 40% of all ED patients [29,30]). The strategic role of emergency services is to identify and monitor the problem right from the beginning, to support planning and coordinate the implementation of interventions, ensuring the presence and competence of trained operators. In view of published data and previous experiences in the Veneto Region and Padova, we studied this phenomenon in order to identify the best methods to track trends and define the procedures performed in the ED, interventions during hospital stays, alarm modes, training of new staff, and communications between operators and the local population. A formal evaluation was made regarding acute alcohol intoxication and alcohol misuse in adolescents and the common consequences of alcoholic behaviors in high school or university students while binge drinking, which has become a more frequent cause of overdoses requiring ambulance services in Italy. Internet sites influence the behavior of adolescents, and serious epidemiological surveillance systems and extra-hospital reports (risk analysis, definition scenarios, anamneses, and organizational schemes, in the case of intoxicated patients admitted to EDs) are critically examined and discussed. These findings have several implications for current clinical practice both outside the hospital and in ED settings; in particular, current findings indicate that we must improve our efforts to reduce the negative impact of this practice on adolescents and focus more on several promising directions for future research. Research teams can provide information and educational support to adolescents, but alcoholic games are often played across different platforms. Studying the path of hospital inpatients or those undergoing observation allowed us to identify categories or types of patients similar in intensity of consumption of resources and clinically significant in relation to the extent of trauma or the cause of hospitalization associated with acute alcohol intoxication. These findings indicate that the cases collected were a substantial source of our better understanding of alcoholic games in order to create successful measures to promote a healthy lifestyle among adolescents. The focus on such an important topic is relatively recent for ED staff. This retrospective study stresses the need for definitive studies with larger sample sizes and a random controlled design. Although our experience is not exhaustive, it does demonstrate the importance of physician recognition and identification of intoxication as a result of alcoholic game competition. We did not aim to be exhaustive but only to give interested readers a specific feeling of progress on the topics necessary to stimulate future researchers in related disciplines in policy-making processes.

### Conclusions

Excessive alcohol use in adolescents and young people (aged 15 to 20 years) who participate in new alcoholic games and are admitted to EDs is associated with negative consequences and continues to be an important health issue, although very little documentation supports our research. Monitoring of changes in alcohol consumption (eg, heavy drinking occasions, drinking traditions, and different social Web patterns) and studying the adverse health consequences of drinking to excess reveal substantial problems, and new methods must be applied to prevent extreme consequences. Different levels of knowledge and channels of communication should help in developing new methods to prevent alcohol abuse in teenagers. The effective communication of the dangers of alcoholic games can help to implement specially designed alcohol education programs in specific contexts to contribute to effecting changes in behavior. The data stress contextual factors and dangerous behaviors due to alcoholic games by adolescent drinkers, but the accidental circumstances of admission to EDs present the opportunity to study the extent of drinking groups, group pressure, and social influences and can also highlight problematic behaviors associated with increased levels of this context-specific and hazardous abuse of alcohol. The new alcoholic games encourage young people to consume large amounts of alcohol quickly, putting them at risk of alcohol poisoning which can potentially cause accidental injuries and contribute to higher levels of unprotected sex, suicide, sexual violence, and traffic accidents. This study shows that the frequency of these events is increasing due to the widespread and often indiscriminate use of social media. Greater awareness is needed to prevent future accidents and grant us deeper understanding of which population subgroups are most at risk in order to establish a defensive and preventive education strategy. Emergency physicians (as well as educators and in general all those who work in health care) have the responsibility to identify and prevent the spread of these patterns of behavior. Health care clinicians should understand the reason why the number of hospitalizations due to alcoholic games has increased; it is the absence of substantial intervention in the educational schooling. We cautiously conclude that our research into the increasing impact of technology on game-related alcohol consumption in adolescents has identified some future steps to development and research, clarifies ED practitioners’ need for more information, and provides recommendations for other health professionals. One key factor for success is to tailor future studies, conducted with epidemiological criteria and extended to other countries, all of which will allow us to understand and analyze the true extent of the problem in order to develop prevention campaigns in hospitals and schools.

## Abbreviations

BAC: blood alcohol content
DRG: diagnosis-related group
ED: emergency department
